# Factors associated with perinatal mortality among public health deliveries in Addis Ababa, Ethiopia, an unmatched case control study

**DOI:** 10.1186/s12884-017-1420-7

**Published:** 2017-07-26

**Authors:** Yemisrach Getiye, Mesganaw Fantahun

**Affiliations:** Addis Ababa University, Addis Ababa, Ethiopia

**Keywords:** Perinatal mortality, Still birth, Early neonatal death

## Abstract

**Background:**

perinatal mortality is the sum of still birth (fetal death) and early neonatal death (ENND) i.e. death of live newborn before the age of 7 completed days. Perinatal mortality accounts three fourth of the deaths of the neonatal period and is one of the major challenges for under-five mortality. Therefore this study was conducted to better understand the common and avoidable factors that affect perinatal mortality in Addis Ababa, Ethiopia.

**Methods:**

An unmatched case control study design using secondary data as a source of information was conducted. Cases were still births or early neonatal deaths and controls were live births and neonates who were discharged alive from the hospital and did not die before the age of 7 days. The study period was from 1st January up to 30th February 2015. Epi-Info version 7.0 and SPSS Version 21 were used for data entry and analysis. Descriptive statistics, frequencies, proportions and diagrams were used to check the distribution of outcome variable and describe the study population. Logistic regression model was used to identify the important factors that are associated with perinatal mortality.

**Results:**

A total of 1113(376 cases and 737 controls) maternal charts were reviewed. The mean age of the mothers for cases and controls were 26.47 ± 4.87 and 26.95 ± 4.68 respectively. Five hundred ninety seven (53.6%) mothers delivered for the first time. Factors that are significantly associated with increased risk of perinatal mortality were birth interval less than 2 years, preterm delivery, anemia, congenital anomaly, previous history of early neonatal death and low birth weight. Use of partograph was also associated with decreased risk of perinatal mortality.

**Conclusion:**

From factors that are associated with perinatal mortality, some of them can be prevented with early investigation of pregnant mothers on their antenatal care follow. Appropriate labor follow-up and monitoring with regular use of partograph, immediate newborn care and interventions to delay birth interval also minimize perinatal mortality.

## Background

Perinatal mortality is total number of deaths in the perinatal period. This includes still birth (fetal death) and early neonatal death (ENND) i.e. death of live newborn before the age of 7 completed days. Perinatal mortality rate (PMR) is calculated as total number of perinatal deaths per total number of births (still births + live births)) × 1000 [1].

Many classification systems have been used to identify the causes of perinatal deaths. According to international classification of disease for perinatal mortality (ICD-PM), there are three distinct features of perinatal mortality: time of a perinatal death in the antepartum, intra partum or neonatal period, cause of death and contributing maternal conditions with perinatal deaths [2].

In developed countries, perinatal mortality is a rare event. According to International Comparisons of Fetal and Neonatal Mortality Rates in High-Income Countries, the range of fetal deaths was from 1.6 to 4.7 per 1000 total births and neonatal deaths was 1.1 to 4.3 per 1000 live births using a birth weight cut-off of 1000 g. Also using gestational age cut-off of 28 weeks, they ranged from 1.7 to 4.9 per 1000 for fetal deaths and 1.3 to 4.0 per 1000 for neonatal deaths [3]. Ninety seven percent of globally reported stillbirths and 98 % of neonatal deaths occurred in developing countries [4].

In 2012, globally 2.9 million newborns died and 2.6 million babies were still born. More than three-quarters of the world’s newborn deaths occurred in South Asia and sub-Saharan Africa, which have both the highest neonatal mortality rates among regions and the largest number of annual births. The region with the largest absolute number of deaths is south Asia and the highest mortality rates are found in sub-Saharan Africa [5]. PMR and intra partum stillbirths are 5 and 14 times higher in developing regions compared with developed regions consecutively [6].

In a poor community, most babies die without a name or unrecorded; it is because of family insecurity about the life of their babies [7]. Ethiopia like other sub-Saharan countries has a high perinatal mortality. According to WHO report, PMR of Ethiopia in 2004 was 41/1000 total births or 128,000 total deaths (34,000 still births and 94, 000 early neonatal deaths) [8]. Based on Ethiopian Demographic and Health survey (EDHS) 2011, PMR is 46/1000 total births [9].

In the past few decades, there has been an impressive decline in the mortality of children under5 years age worldwide. However, the rate in reduction of neonatal mortality is much slower and by 2012, the share of neonatal mortality for under5 mortality is 44% [10]. Perinatal mortality contributes for three fourth of the deaths of the neonatal period and the day the baby is born is the most dangerous time for the newborns life [11]. Therefore assessing factors that are associated with perinatal mortality or factors that predispose to causes of perinatal mortality is essential to minimize neonatal mortality [8].

There are limited numbers of studies done related to perinatal deaths in our country including Addis Ababa. *Therefore the purpose of this study was to assess the socio demographic, obstetric, medical,* newborn and health care related factors associated with perinatal mortality in Addis Ababa, Ethiopia.

## Methods

A hospital based unmatched case control study design using secondary data as a source of information was conducted in four public hospitals of Addis Ababa: Tikur Anbessa, Gandhi memorial, Zewditu memorial and St. Paul hospitals. These hospitals were selected based on availability of both delivery and neonatal intensive care unit service during the time of data collection. All these hospitals serve as a referral and teaching hospital both at city administration and federal level. All deliveries conducted from January 1/ 2014-Dec 31/ 2014 were used as a source population. Cases were still births and early neonatal deaths while controls were newborns delivered alive and did not die before the age of 7 days.

The sample size was calculated by considering different factors that are strongly associated with perinatal mortality according to a study done on 2008 in Addis Ababa: age, parity, low birth weight, antenatal care follow up and past history of perinatal death. Based on the assumption of case to control ratio of 1:2, 95% confidence level, Power of 80% and odds ratio of 2 and taking maximum sample size, the total sample size for this study became 1128(376 cases 752 controls). The Sample size was assigned to the study hospitals proportionally based on average delivery rate of three months prior to data collection. Then from 2014 delivery report, using delivery and neonatology log books; cases were systematically selected. For each case, 2 controls delivered in the same day as of cases were selected considered as a control group and then a phone call was conducted to prove that discharged neonates were alive up to seven completed days. For a neonate discharged alive and died before seven completed days the next alive neonate was taken as a control group. The mother was taken as a non-respondent if they didn’t respond to a phone call or if they are not available.

Data was collected using structured questionnaire from medical records of mothers and neonates. Admission history, labor follow up sheet, delivery summary and antenatal care (ANC) follow up sheet were used. The dependent variable is perinatal mortality and the independent variables are Socio demographic factors, obstetric factors, medical factors, newborn factors and health care factors. Ethical clearance was obtained from Research ethics committee (REC) of School of Public Health in Addis Ababa University (AAU), Addis Ababa health bureau and hospitals respectively. Since this study is from medical records except one question that was responded by phone call, informed verbal consent was obtained during a phone call from each selected participants. Furthermore confidentiality was assured during data collection, analysis and dissemination of the result.

The data were coded, checked and entered using Epi-Info version 7.0 and exported to SPSS Version 21 for analysis. Descriptive statistics and cross tabulation were used to describe the study population. Bivariate logistic regression analysis was used to assess the degree of association between dependent and independent variables and test significance of the association. Odds ratio with 95% confidence interval was used to measure strength of association. Those variables associated at bivariate logistic regression with significance level of *p* value <0.2 were entered into multivariate logistic regression model to identify the important determinants by controlling possible confounding effects.

## Result

Demographic and obstetric characteristics of mothers of cases and controls: A total of 1113(376 cases and 737 controls) maternal charts were reviewed, with a response rate of 98.7%. The mean age of the mothers for cases and controls was 26.47 ± 4.87 and 26.95 ± 4.68 respectively. Of the total respondents, 597(53.6%) delivered for the first time. From 516 mothers who had previous history of delivery, 24.1% of cases and 8.4% of controls delivered within two years of previous delivery. More than 95% of mothers had regular ANC follow up and took Tetanus Toxoid (TT) vaccination during current pregnancy. The proportion of preterm delivery was 37.0% in cases and 8.0% in controls. More than half (58.5%) of the mothers were delivered by Spontaneous Vertex Delivery (SVD). From the total respondents, 5.3% and 2.9% of cases and 3.3% and 0.7% of controls had previous history of stillbirth and early neonatal death respectively (Table 1).

**Table 1:** Demographic and obstetric characteristics of mothers of perinatal deaths (cases) and controls among public hospital deliveries in Addis Ababa, Ethiopia. 2014

category		PERINATAL OUTCOME		Total
		control	case	
Maternal age	15–19	40(5.4%)	16(4.3%)	56(5.0%)
	20–24	213(28.9%)	96(25.5%)	309(27.8%)
	25–29	289(39.2%)	147(39.1%)	436(39.2%)
	30–34	135(18.3%)	87(23.1%)	222(19.9%)
	≥35	60(8.2%)	30(8.0%)	90(8.1%)
Parity	1	391(53.1%)	206(54.8%)	597(53.6%)
	2–4	330(44.8%)	163(43.4%)	493(44.3%)
	≥5	16(2.2%)	7(1.9%)	23(2.1%)
Birth interval	<2 years	29(8.4%)	41(24.1%)	70(13.6%)
	≥ 2 years	256(74%)	69(40.6%)	325(63.0%)
	unknown	61(17.6%)	60(35.3%)	121(23.4%)
ANC follow up	yes	724(98.2%)	354(94.1%)	1078(96.9%)
	No	13(1.8%)	22(5.9%)	35(3.1%)
TT vaccination	Yes	714(96.9%)	348(92.6%)	1062(95.4%)
	No	12(1.6%)	18(4.8%)	30(2.7%)
	unknown	11(1.5%)	10(2.7%)	21(1.9%)
Gestational age	37–41	572(77.6%)	188(50.0%)	760(68.3%)
	28–36	59(8.0%)	139(37.0%)	198(17.8%)
	>42	83(11.3%)	23(6.1%)	106(9.5%)
	unknown	23(3.1%)	26(6.9%)	49(4.4%)
Mode of delivery	SVD	404(54.8%)	247(65.7%)	651(58.5%)
	Instrumental^a^	55(7.5%)	11(2.9%)	66(5.9%)
	C/S	272(36.9%)	73(19.4%)	345(31.0%)
	ABD	6(0.8%)	27(7.2%)	33(3.0%)
	Hysterectomy	0(0.0%)	18(4.8%)	18(1.6%)
Previous history of stillbirth	Yes	24(3.3%)	20(5.3%)	44(4.0%)
	No	713(96.7%)	356(94.7%)	1069(96.0%)
Previous History of ENND	Yes	5(0.7%)	11(2.9%)	16(1.4%)
	No	732(99.3%)	365(97.1%)	1097(98.6%)

^a^forceps and vacuum delivery

*SVD* Spontaneous Vertex Delivery, *C/S* cesarean section, *ABD* Assisted Breach Delivery

Coexisting medical and obstetric complications and newborn characteristics: Hemoglobin was determined for 74.2% of mothers during ANC follow up or before delivery. Anemia was presented in 15.4% of cases and 8.0% of controls. The proportion of mothers who have chronic illnesses was almost similar within the two groups of population; 5.6% in cases and 5.0% in controls. Two hundred forty two (64.4%) mothers of the cases and 323(43.8%) mothers of the controls had at least one type of obstetric complication. From all obstetric complications, preeclampsia and preterm labor accounts for 34.3% and 43% of cases and 21.7% and 13% of controls respectively. Concerning the type of delivery 95.8% of mothers delivered a single newborn with comparable proportion between cases and controls. The mean birth weight of newborns was 2855 ± 724 g with a minimum of 1000 g and maximum of 4600 g. Very low birth weight and low birth weight newborns were more common in cases, 18.1% and 36.2% respectively. Congenital anomaly occurred 20 times more in cases than controls (Table 2).

**Table 2:** Coexisting medical, obstetric complications and newborn characteristics of perinatal deaths (cases) and controls among public hospital deliveries in Addis Ababa, Ethiopia. 2014

category		PERINATAL OUTCOME		Total
		control	case	
Level of hemoglobin	<11 g/dl	41(15.4%)	45(8.0%)	86(10.4%)
	≥11 g/dl	225(84.6%)	515(92.0%)	740(89.6%)
Obstetric complications	Yes	242(64.4%)	323(43.8%)	565(50.8%)
	No	134(35.6%)	414(56.2%)	548(49.2%)
Fetal Presentation	Cephalic	680(92.3%)	333(88.6%)	1013(91.0%)
	Malpresentation*	57(7.7%)	43(11.4%)	100(9.0%
Type of delivery	Single	708(96.1%)	358(95.2%)	1066(95.8%)
	Multiple	29(3.9%)	18(4.8%)	47(4.2%)
Weight	2500-3999 g	655(88.9%)	161(42.8%)	816(73.3%)
	1000-1499 g	0(0.0%)	68(18.1%)	68(6.1%)
	1500-2499 g	45(6.1%)	136(36.2%)	181(16.3%)
Congenital anomaly	Yes	3(0.4%)	60(16.0%)	63(5.7%)
	No	734(99.6%)	316(84.0%)	1050(94.3%)

Malpresentations* shoulder, brow or face presentation

Health care related factors: Partograph was used in 816(73.3%) of total study subjects. The proportion of mothers who delivered without partograph follow up was 79(21.0%) in cases and 66(9.0%) in control groups. From two hundred sixty five stillbirths, 57 (21.5%) of them were admitted to the hospital with positive fetal heart beat and later reported as stillbirth during the course of labor and delivery. From early neonatal deaths, majority (55%) of the neonates’ first minute APGAR score was low (4–6). While looking fifth minutes APGAR score, 47.7% of neonates APGAR score was very low (0–3).

Factors associated with perinatal mortality: Binary logistic regression was done to assess the association of perinatal mortality with different characteristics. Both crude and adjusted analysis showed that birth interval, gestational age, mode of delivery, previous history of ENND, level of hemoglobin, newborn weight, congenital anomaly and use of partograph are significantly associated with perinatal mortality. Among obstetric characteristics of the mother, the odds of perinatal mortality were 4.6 times higher among mothers whose previous delivery was within two years of current delivery compared to mothers whose previous delivery was more than or equal to two years back(AOR 4.55; 95% CI (1.79–11.54)). The odds of perinatal mortality were two times higher among preterm deliveries than term deliveries (AOR 2.02; 95%CI (1.08–3.77)). Considering mode of delivery, the odds of perinatal mortality were less likely among mothers who delivered by instrument or cesarean section than mothers who delivered by SVD with AOR0.21; 95% CI (0.05–0.86) and AOR 0.48; 95%CI (0.27–0.86) respectively. The odds of perinatal mortality were also higher among mothers who had previous ENND than mothers with no previous history of ENND (AOR 6.36; 95%CI (1.51–26.76)). Among coexisting medical conditions, perinatal mortality were 2.6 times more likely among anemic mothers than mothers with normal hemoglobin level (AOR 2.6; 95%CI (1.38–4.91)). Based on newborn characteristics, the odds of perinatal mortality were higher among low birth weight newborns than normal weighing newborns (AOR 16.45; 95%CI (9.57–28.26)) and the odds of perinatal mortality for newborns with congenital anomaly were higher than newborns with no congenital anomaly (AOR 34.04; 95%CI (7.14–162.41)). Use of partograph found to be a protective factor for perinatal mortality. The odds of perinatal mortality were 65% less likely among mothers whose labor was monitored using partograph compared with mothers whose labor is not monitored by partograph (AOR 0.35; 95%CI (0.18–0.66)) (Table 3).

**Table 3:** The association between different factors and perinatal mortality among public hospital deliveries in Addis Ababa, Ethiopia, 2014

variables	category	Perinatal outcome		Crude odd ratio (95% CI)	Adjusted odd ratio (95% CI)
		case	control		
Educational status	Not educated	48(12.8%)	55(7.5%)	1	1
	primary	85(22.6%)	128(17.4%)	0.76(0.47–1.22)	0.82(0.37–1.81)
	Secondary &above	202(53.7%)	455(61.7%)	0.51(0.33–0.76)*	0.54(0.27–1.11)
	unknown	41(10.9%)	99(13.4%)	0.48(0.28–0.81)*	0.39(0.16–0.98)
Birth interval	≥2 Years	69(18.4%)	256(34.7%)	1	1
	<2 Years	41(10.9%)	29(3.9%)	5.25(3.04–9.05)*	4.55(1.79–11.54)*
	unknown	60(16.0%)	61(8.3%)	3.65(2.34–5.69)*	2.62(1.14–6.01)*
ANC follow up	Yes	354(94.1%)	724(98.2%)	1	1
	No	22(5.9%)	13(1.8%)	3.46(1.72–6.95)*	6.15(0.31–122.04)
TT vaccination	Yes	348(92.6%)	714(96.9%)	1	1
	No	18(4.8%)	12(1.6%)	3.08(1.47–6.46)*	0.19(0.01–4.70)
Gestational age	Term	188(50.0%)	572(77.6%)	1	1
	Preterm	139(37.0%)	59(8.0%)	7.168(5.07–10.13)*	2.02(1.08–3.77)*
	Post term	23(6.1%)	83(11.3%)	0.84(0.52–1.38)	1.16(0.57–2.35)
	unknown	26(6.9%)	23(3.1%)	3.44(1.92–6.17)*	2.63(0.95–7.26)
Mode of delivery	SVD	247(65.7%)	404(54.8%)	1	1
	Instrumental	11(2.9%)	55(7.5%)	0.33(0.17–0.64)*	0.21(0.05–0.86)*
	Cesarean Section	73(19.4%)	272(36.9%)	0.44(0.32–0.59)*	0.48(0.27–0.86)*
	Breach delivery	27(7.2%)	6(0.8%)	7.36(2.99–18.08)*	1.94(0.47–8.03)
	Hysterectomy	18(4.8%)	0(0.0%)	-	-
history of ENND	No	365(97.1%)	732(99.3%)	1	1
	Yes	11(2.9%)	5(0.7%)	4.41(1.52–12.79)*	6.36(1.51–26.76)*
Level of hemoglobin	≥11 g/dl	225(84.6%)	515(92.0%)	1	1
	< 11 g/dl	41(15.4%)	45(8.0%)	2.09(1.33–3.28)*	2.6(1.38–4.91)*
Obstetric complications	No	134(35.6%)	414(56.2%)	1	1
	Yes	242(64.4%)	323(43.8%)	2.32(1.79–2.99)*	1.38(0.84–2.27)
Fetal presentation	vertex	333(88.6%)	680(92.3%)	1	1
	Mal presentation	43(11.4%)	57(7.7%)	1.54(1.02–2.34)*	1.52(0.65–3.59)
Weight of the newborn	2500-3999 g	161(42.8%)	655(89.9%)	1	1
	<2500 g	204(54.3%)	45(6.1%)	18.44(12.79–26.59)*	16.45(9.57–28.26)*
	≥4000 g	11(2.9%)	37(5.0%)	1.210(.604–2.423)	2.24 (0.89–5.67)
Congenital anomaly	No	316(84.0%)	734(99.6%)	11	1
	Yes	60(16.0%)	3(0.4%)	46.46(14.46–149.23)*	34.04(7.14–162.41)*
Partograph use	No	79(21.0%)	66(9.0%)	1	1
	Yes	252(67.0%)	564(76.5%)	0.37(0.26–0.54)*	0.35(0.18–0.66)*
	Not Indicated	45(12.0%)	107(14.5%)	0.35(0.22–0.57)*	0.37(0.14–0.98)*

*- *p*-value <0.05

## Discussion

Of the factors associated with perinatal mortality, birth interval less than two years is 4.5 times high likely among perinatal death (AOR 4.55; 95% CI(1.80–11.54)). This is consistent with findings in different parts of the country and might be explained by short birth interval increases the risk of obstetric complications that can affect the perinatal outcome [12–14]. In this study parity didn’t show an association with perinatal death. This is also supported by a study done in Hawassa university hospital of Ethiopia [12]. However, a study done in Democratic Republic Congo found primi parity and studies done in eastern Sudan and Nigeria showed parity > 4 increase the risk of perinatal death [15–17]. The difference in the association might be because of difference in the study design, study populations or sample size. Mothers with previous history of ENND were independently associated with perinatal death in current pregnancy. This is similar to a study done on past reproductive performance and its correlation with perinatal outcome which was done in teaching hospitals of Addis Ababa [14], in which the risk of perinatal death was 10.6 times higher when there was previous history of perinatal loss. The study also identified that anemic mothers were 2.6 times more likely to have perinatal death compared to mothers with normal hemoglobin level. This can be explained by the fact that anemia can increase maternal morbidities and poor pregnancy outcome including perinatal mortality [18]. According to a study done in Hawassa university hospital of Ethiopia, obstetric complications mainly obstructed labor were strongly associated with perinatal mortality with a case fatality rate of 73.5% [12] but this is not supported by this study. This might be because of low proportion of obstructed labor (0.9% in controls and 2.5% in cases) in this study and the difference in the study setting. The other reason is may be because of skilled health professionals who can identify obstructed labor and manage them accordingly. Based on the findings of this study, perinatal mortality was 2 times more likely among preterm newborns compared with term newborns. This is similar with study finding done in Hawassa university hospital in which perinatal mortality was 3 times higher among preterm babies than term babies [12]. The association of preterm babies with perinatal mortality is mainly because of prematurity and save the children report shows that one of the commonest causes of perinatal death is preterm birth and its complications [5]. Low birth weight newborns had also a high risk for perinatal mortality than normal weight babies. The weight of newborns was related with gestational age. In which 26.8% of preterm deliveries were very low birth weight and 41.9% were low birth weight. This finding is comparable with different researches done in Ethiopia and other African countries [13, 19]. Congenital anomaly had poor outcome for newborns. Perinatal mortality was much higher among newborns with congenital anomaly (AOR 34.04; 95%CI (7.14–162.41)), which is similar with a study done in Zimbabwe that showed 5 times risk of perinatal death [19]. Large odds ratio with wide confidence interval could be due to small number of congenital anomalies found in the control groups with a case control ratio of 20:1 or the sample size may not be enough. Use of partograph was protective factor for perinatal death by 65%. This is supported by a study done on perinatal death audit in peri-urban hospital in Kampala, Uganda [20]. Since partograph is a labor follow up chart recommended by WHO, appropriate use of partograph can help health professionals to pick any abnormalities during the course of labor. Therefore it can prevent perinatal loss with early diagnosis and management of labor complications. From stillbirth found in this study, majority (78.5%) of them were admitted to the hospital with negative fetal heart beat. This is supported by systematic review done from sixteen hospitals and community based perinatal mortality studies [21]. Even though there was a large amount of stillbirth occurred before arrival to hospital, 21.5% of stillbirths occurred during intra partum period that can be averted with appropriate intra partum care. For newborns who delivered alive with low APGAR score, appropriate and timely immediate newborn care is mandatory to save their lives. This study showed that large numbers of newborns first minute APGAR score was within range of 4–6 but in the fifth minute the majority fall in the range of 0–3. This tells that the newborn care and resuscitation is not satisfactorily effective to save newborn lives. According to the finding of this study perinatal mortality was less likely among mothers who delivered by instruments (forceps or vacuum) or cesarean section than SVD. Since this kind of delivery was commonly conducted when there was an obstetric complication and when adjusting obstetric complications for mode of delivery, the association didn’t occur. The finding of a case control study in Marondera district, East of Zimbabwe in 2009 also showed that normal SVD is a protective factor for perinatal death compared with instrumental delivery or cesarean section [19].

The main limitation of this study is, it has used secondary data as a source of information. Since this data was gathered for other purpose, it was difficult to gather all necessary variables and some variables were not found some of the charts. Therefore the confounding effect of unmeasured variables cannot be controlled. This may affect the association of outcome variable and the exposure.

## Conclusion

Of the factors identified as associated with perinatal mortality; birth interval less than two years, preterm delivery, previous history of ENND, anemia, low birth weight and congenital anomaly increases the risk of perinatal mortality and use of partograph for labor follow up decreases the risk of perinatal mortality. From factors that are mentioned above, some of them can be prevented with early investigation of pregnant mothers up on their follow up to identify abnormalities and manage them accordingly. The other factor is poor quality of intra partum care, reflected by not using partograph for labor follow up which is one of the important determinant factors for perinatal loss. Optimizing immediate newborn care and neonatal resuscitation can also avert lots of early neonatal deaths.

## Abbreviations

AAU: Addis Ababa University
ANC: Antenatal Care
AOR: Adjusted Odd Ratio
C/S: Cesarean section
EDHS: Ethiopian Demographic and Health Survey
ENND: Early Neonatal Death
PMR: Perinatal Mortality Rate
REC: Research Ethics Committee
SVD: Spontaneous Vertex Delivery
TT: Tetanus Toxoid
WHO: World Health Organization

## References

[1] USAID. Measure evaluation population and reproductive health, perinatal mortality rate. https://www.measureevaluation.org/prh/rh_indicators/womens-health/nb/perinatal-mortality-ratepmrand reproductive health. Accessed Aug 2014.

[2] Allanson E, Tunçalp Ö, Gardosi J, Pattinson R, Jaap J, Flenady VJ (2016). Classifying the causes of perinatal death. Bull World Health Organ.

[3] Ashna D, mail M, Blondel B, Gissler M, Velebil P, Macfarlane A, et al. International Comparisons of Fetal and Neonatal Mortality Rates in High-Income Countries: Should Exclusion Thresholds Be Based on Birth Weight or Gestational Age? PLoS ONE. 2013;8(5). 10.1371/journal.pone.0064869 PMC3658983 23700489

[4] Zupan, J. Perinatal mortality in developing countries*.* J Med. May 19, 2005; 352: 2047-2049. 10.1056/NEJMp058032 15901857

[5] Save the children. *Healthy newborn*. 2009.http://www.healthy newborn network.org. Accessed 11 Aug 2014.

[6] WHO, neonatal and perinatal mortality: country, regional and global estimate: Geneva, Swetherland. 2006.

[7] Joy E Lawn, Simon Cousens and Jelka Zupan. *4 million neonatal deaths: when?where?why?*. The lancet March 2005; 365: p. 891–900. 10.1016/S0140-6736(05)71048-5 15752534

[8] Ahman E, Zupan J. Neonatal and perinatal mortality : country , regional and global estimate 2004. Geneva: World Health Organization; 2007.

[9] Central Statistics Agency, Ethiopia and ICF international. Ethiopian demographic and health survey. Addis Ababa, Ethiopia. 2012.

[10] Peter Cooper. *Strategies to reduce perinatal mortality.* The lancet global health.2016; 4(1): 6–7. 10.1016/S2214-109X(15)00268-5 26639856

[11] WHO. *Every new born: an action plan to end preventable deaths.* February 2014.

[12] Bayou G, Berhan Y (2012). Perinatal mortality and associated risk factors, a case control study. Ethiop J Health Sci.

[13] Andarge G, Birhan Y. Alemayehu Worku and Yigaw kebede. *peridictors of perinatal mortality in rural population of north west Ethiopia: prospective longitudinal study*. BMC Public Health. 2013;13 10.1186/1471-2458-13-168 PMC3599850 23433304

[14] Tilahun S, Gaym A (2008). Past reproductive performance and its correlation with perinatal mortality among delivering mothers at Addis Ababa teaching hospital. Ethiop Med J.

[15] Ntambue AM, Donnen P, Dramaix-Wilmet M, Malonga FK (2012). Risk factors for perinatal mortality in the city of Lubumbashi. Revue Epidemiol et de santé publique.

[16] Ali AA, Elgessim ME, Taha E, Adam GK (2014). Factors associated with perinatal mortality.kassala,Eastern sudan. J Trop Pediatr.

[17] Onadeko MO, Asekun-Olarimoye EO, Lawoyin TO (2010). Neonatal mortality and perinatal risk factors in rural southwestern Nigeria: a community based prospective study. WestAfrican J Med.

[18] Allen LH. Am J Clin Nutr. Anemia and iron deficiency: effects on pregnancy outcome, 2000;71(5). 10.1093/ajcn/71.5.1280s 10799402

[19] El T, Gombe N, Shambira G, Chadambuka A, Mufuta T, Zizhou S. Determinants of perinatal mortality in Marondera district, Mashonal and East Province of Zimbabwe.2009. Pan AFr Med J. 2011;8(7). 10.4314/pamj.v8i1.71054 PMC3201615 22121416

[20] Nakibuuka VK, P. (2012). Okong, and RN Byaruhang. *Perinatal death audits in a peri-urban hospital in Kampala, Uganda*. J Afr Health Sci.

[21] Berhan Y, Berhan A (2014). Perinatal Mortality Trends in Ethiopia. Ethiop J Health Sci.

